# A volume-conformity paradoxon: Volumetric determinants of treatment success/failure following stereotactic radiosurgery in vestibular schwannoma

**DOI:** 10.1093/noajnl/vdag139

**Published:** 2026-05-27

**Authors:** Sophie Shih-Yüng Wang, Georgios Naros, Albertus van Eck, Marcos Tatagiba, Gerhard Horstmann

**Affiliations:** Department of Neurosurgery, University Hospital Tübingen, Eberhard Karls University, Tübingen, Germany; Department of Neurosurgery, University Hospital Tübingen, Eberhard Karls University, Tübingen, Germany; Gamma Knife Center Krefeld, Krefeld, Germany; Department of Neurosurgery, University Hospital Tübingen, Eberhard Karls University, Tübingen, Germany; Gamma Knife Center Krefeld, Krefeld, Germany

**Keywords:** radiosurgery, skull base, vestibular schwannoma

## Abstract

**Background:**

Stereotactic radiosurgery (SRS) is an established, minimally invasive treatment for vestibular schwannoma (VS). However, tumor control rates vary, and predictors of treatment failure remain debated. This study examines whether pre-treatment tumor volume (TV) predicts SRS outcome and explores the relationship between tumor size, Paddick Conformity Index (PCI), and long-term control.

**Methods:**

We retrospectively analyzed 928 consecutive patients with solitary VS treated using Gamma Knife radiosurgery. Patients with <2 years of radiographic follow-up were excluded to avoid pseudoprogression. Tumor size was classified using the Koos system, and volumetric measurements were obtained via gadolinium-enhanced magnetic resonance imaging. Treatment failure was defined as sustained volumetric progression. Predictive performance was evaluated using receiver operating characteristic and multivariate analysis comparing TV, Koos class, and PCI.

**Results:**

Mean follow-up was 6.4 ± 4.0 years. The overall recurrence rate was 10%, varying by Koos class (I: 4%; II: 10%; III: 13%; IV: 10%; *P* = .017). PCI increased with tumor size but showed reduced predictive accuracy in larger tumors (area under the curve [AUC] 0.69 for Koos I vs. 0.48 for Koos IV). TV and KOOS classification yielded comparable predictive performance (AUC 0.57 and 0.60). In a multivariate analysis, neither TV, KOOS classification, nor PCI were independent predictors of treatment failure, whereas sex remained significantly associated with progression.

**Conclusions:**

Pre-treatment TV is associated with radiosurgical outcome in VS. Increasing tumor size correlates with higher recurrence risk and reduced reliability of conformity indices—constituting a volume-conformity paradoxon with implications for individualized treatment planning, particularly for large tumors.

Key Points

- The rate of treatment failure increases with increasing pre-treatment tumour size and volume after stereotactic radiosurgery (SRS) in vestibular schwannoma (VS).
- Paddick Conformity Index’s performance to predict treatment failure decreased in increasing tumor volume.
- Treatment strategy should be adapted in large VS, which represent a heterogenous group, in order to improve treatment response after *SRS*. If tumor responded to SRS (treatment success), the rate of long-term postinterventional volume reduction was the highest in large tumors.

Importance of the Study

The factors influencing treatment success or failure after stereotactic radiosurgery (SRS) for vestibular schwannoma (VS) remain an area of ongoing investigation. Previous studies have reported inflicting outcomes in relation to tumor size. This study, based on one of the largest single-institution cohorts with long-term follow-up, provides robust evidence that pretreatment tumor volume (TV) is a significant predictor of treatment failure rate following SRS. Moreover, Paddick Conformity Index’s performance to predict treatment failure decreased with increasing tumor size. If large tumors present responsive to treatment, their rate of TV reduction is the highest. By providing volumetric benchmarks for clinical decision-making, this study adds critical nuance to the current understanding of radiosurgical outcomes in VS and has direct implications for patient selection, counseling, and protocol development.

Vestibular schwannoma (VS) represent the most common intracranial neoplasm in the cerebellopontine angle.^1^^,^^2^ According to several treatment guidelines and recommendation stereotactic Radiosurgery (SRS) is a valid noninvasive treatment option in VS.^1^^,^^2^ However, studies have shown that a subset of patients demonstrate radiological progression following SRS (treatment failure).^3-6^ Identifying reliable pretreatment predictors of treatment failure is therefore crucial to improving patient selection, optimizing therapeutic planning, and refining outcome expectations. Female sex, cystic morphology and postinterventional new-onset-facial spasm have been described to be associated with lower rates of treatment success.^4-6^ Among the various prognostic factors proposed, tumor volume (TV) at the time of treatment has gained increasing attention.^7-9^

Tumor size can be classified in multiple ways with their own advantage and disadvantage in methodology: through size classification (e.g., KOOS or Hannover classification), tumor diameter, or tumor volumetry.^10-14^ While tumor size significantly affects postinterventional functional outcomes after surgery, the literature presents conflicting views on its role as a predictor of treatment success after SRS in VSs.^2^^,^^3^ While several studies have suggested that larger tumor size may negatively influence radiosurgical outcomes, others have reported favorable tumor control in large tumors or even linked smaller tumor size with higher rates of treatment failure.^14-23^ The Paddick Conformity Index (PCI) provides a standardized quantitative measure of this parameter and enables investigation of whether increasing TV is associated with reduced conformity, a factor that could account for differences in tumor control across size categories.^24^ Given the substantial influence of TV on radiosurgical planning and dose distribution, evaluation of the PCI represents a crucial component of comprehensive dosimetric assessment and treatment success.

Given the heterogeneity of findings and the clinical importance of identifying non-responders early, further high-quality data are needed to clarify the prognostic value of pretreatment TV. This study aims to evaluate the predictive value of initial TV on treatment outcomes in a large cohort of patients with solitary VSs treated with SRS. By analyzing volumetric, conformity, and clinical data over an extended follow-up period, we seek to determine whether tumor size and TV correlates with treatment success or failure and to inform future stratification strategies for patients undergoing radiosurgical intervention.

## Methods

### Study Design

This is a retrospective cohort study. Patients were identified by a prospectively kept registry. Some clinical data was then retrospectively collected. All VS patients in the SRS cohort received Gamma-Knife-Radiosurgery (GKS) (Leksell Gamma Knife Type B from 1998-1999, Leksell Gamma Knife Type 4C from 1999 to 2009, and Leksell Gamma Knife Perfexion from 2009 to 2019- Elekta AB, Stockholm, Sweden) with a prescription dose of 12-13 Gy (Gray) to the 65% isodose line. All consecutive patients with solitary VS and GKS treatment were screened for this study. Exclusion criteria were (1) bilateral VS, (2) known Neurofibromatosis, (3) loss of follow-up or follow-up less than two years, and (4) previous treatment with either surgery or SRS. The PCI was calculated as follows^24^:


$$Paddick\;Conformity\;Index\left(PCI\right)=\frac{TV_{PIV}{\;}^{2}}{TV\;*\;PIV}$$

TV = Tumor volume

TV_PIV_ = target volume covered by the prescription isodose volume

PIV = prescription isodose volume

The local ethics committee approved this analysis and was according to the ethical standards laid down in the Declaration of Helsinki for research involving human subjects. Study reporting followed the Strengthening the Reporting of Observational Studies in Epidemiology guidelines.

### Data Collection and Volumetric Measurements

**Figure 1.**
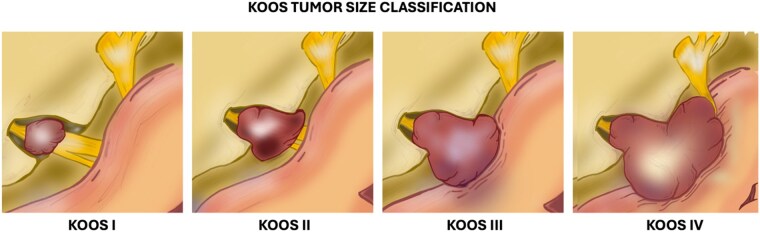
KOOS classification: This figure shows the KOOS classification. Adapted from Koos et al.^10^

Tumor size was classified by Koos classification (Figure 1). Recurrence-free-survival (RFS) was assessed radiographically by gadolinium enhanced magnetic resonance imaging (MRI).^17^^,^^25^ Tumor volumetry was carried at each follow-up timepoint using slice-by-slice manual contouring (slice thickness ≤3 mm) using the software available on the planning software of the treatment device. The criterion for tumor progression was progredient growth after 2 years in gadolinium contrast-enhanced MRI (radiographic tumor control [RTC]) and was also judged by two board-certified neurosurgeons with high VS-related radiosurgery expertise (GH and AvE). Therefore, treatment outcome was defined based on volumetric response, whereas Koos classification was used for anatomical size stratification and descriptive clinical comparison. Functional hearing was defined as Gardner-Robertson 1-2, while functional facial function was assessed by House-Brackman. House-Brackman grades 1-2 was considered good facial function.^25^^,^^26^

### Statistical Analysis

Statistical analysis was performed in R Studio (Version 1.2) using descriptive statistics. To compare nonnumeric parameters of both groups, the chi-square test was applied. For numeric parameters, Welch’s two sample *t*-test was used. RFS was estimated using the Kaplan-Meier method and compared between cases and controls using a log-rank test. The length of follow-up for RFS was calculated from the date of radiosurgical intervention to the date of either recurrence or the last clinical visit. Significance was defined as the probability of a two-sided type 1 error being <5% (*P* < .05). Data are presented as mean ± standard deviation (SD) if not indicated otherwise.

The discriminative performance of different regression models for predicting treatment failure were evaluated using KOOS-classification, TV and PCI. Receiver operating characteristic (ROC) curves were constructed, and the area under the curve (AUC) was used as a measure of model discrimination. AUCs were compared using DeLong’s test for correlated ROC curves.

To identify independent predictors of progression, a multivariable Cox proportional hazards regression model was constructed including log-transformed TV, age, sex, and the PCI. Proportional hazards assumptions were assessed and fulfilled.

## Results

### Patient Cohort

**Figure 2.**
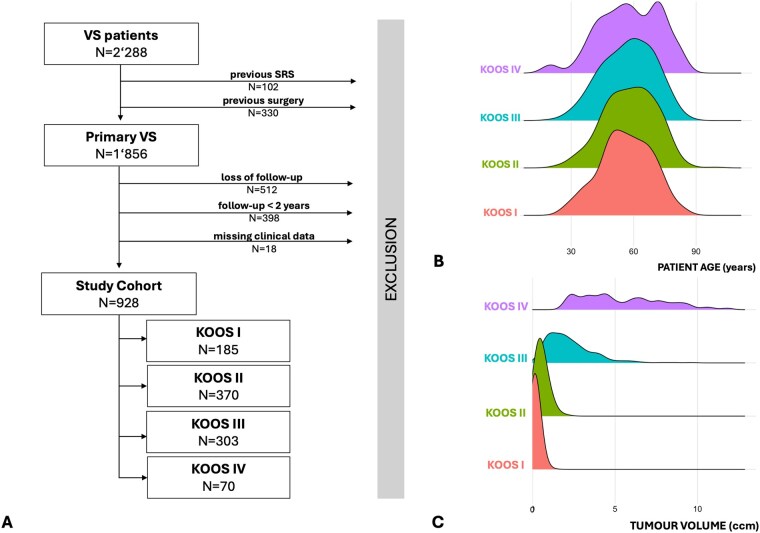
Overall patient cohort, age and tumor volume distribution: (A) shows a flowchart with the study cohort including the number of patients and reason excluded from this study. (B) Shows a ridge plot of the age distribution and frequencies dependent on the KOOS subgroup. (C) Shows the tumor volume distribution in the KOOS subgroups.

The study cohort included *N *= 928 VS patients treated with GKR between 1998 and 2019, who met the above-mentioned inclusion criteria. A flowchart of the study cohort is shown and the tumor size distribution according to the KOOS classification in Figure 2. The majority of the study cohort was female with 57%. The female majority was also present in all KOOS subgroups with 62% of females in KOOS I, 53% in KOOS II, 57% in KOOS III, and 59% in KOOS IV. Mean age was 57.58 (±12.67) years of the overall study cohort.

Demographics are shown in Table 1. The rate of female patients was significantly higher in the treatment failure subgroup (*P = .032*). The age distribution is shown in Figure 2B. Mean TV was 1.38 (±1.80) ccm in the overall study cohort—with a mean TV in KOOS I of 0.16 (±0.13) ccm, 0.54 (±0.34) ccm in KOOS II, 2.23 (±1.43) ccm in KOOS III, and 5.45 (±2.48) ccm in KOOS IV. The tumor size variability, which is largest in KOOS IV tumors are shown in Figure 2C. The volume in ccm is the most heterogenous in groups KOOS III and IV. PCI was significantly higher in the treatment failure group (Table 1).

**Table 1.** Patient demographics and Paddick Conformity Index

	All (*N* = 928)	Treatment success (*N* = 837)	Treatment failure (*N* = 91)	*P*
Age (Years)	57.58 (±12.67)	57.70 (±12.80)	56.50 (±11.47)	.391
Female	524 (56)	463 (55)	61 (67)	.032*
Tumor volume (ccm)	1.38 (±1.80)	1.37 (±1.81)	1.51 (±1.70)	.481
Tumor size				
Koos I	185 (20)	177 (21)	8 (9)	.004*
Koos II	370 (40)	333 (40)	37 (41)	.343
Koos III	303 (33)	264 (32)	39 (43)	.034*
Koos IV	70 (7)	63 (7)	7 (7)	1
Paddick Conformity Index	0.79 (±0.15)	0.78 (±0.16)	0.82 (±0.07)	.018*

Values are presented as the number of patients (%) unless indicated otherwise.

* *P* < .05.

### Functional Status and Postinterventional Outcome

Good facial function was present in 98% of all patients at time of treatment (KOOS I: 99%, KOOS II: 98%, KOOS III: 98%, KOOS IV: 97% [*P =* .772]). Facial function preservation was present in 99% in KOOS I, 99% in KOOS II, 99% in KOOS III, and 99% in KOOS IV (*P= .578*). Functional hearing was present in 53% in the overall cohort and was decreasing according to tumor size classification (KOOS I: 63%, KOOS II: 56%, KOOS III: 48%, and KOOS IV: 32%; [*P <* .001]). Functional long-term preservation in terms of hearing after SRS was 39% in KOOS I, 49% in KOOS II, 50% in KOOS III and 48% in KOOS IV (*P =* .081) at last follow-up.

### Treatment Response in Regard to Tumor Control

Mean follow-up time was 6.37 (±3.96; range 2-21) years in the overall study cohort with 5.68 (±3.42; range 2-18) years in KOOS I, 5.90 (±3.48; range 2-17) years in KOOS II, 6.84 (±4.30; range 2-21) years in KOOS III and 8.57 (±5.03; range 2-19) years in KOOS IV. The rate of treatment failure (recurrence rate) was 10% (*N* = 91/928) in the overall study cohort. Of these, 14% received second SRS treatment, 4% received a ventriculoperitoneal shunting and 81% were referred to a tertiary neurosurgical center for microsurgical treatment. The rate of treatment failure was significantly different depending on KOOS classification (*P =* .017*) (Table 2).

**Table 2.** Parameters depending on KOOS classification

	KOOS I (*N* = 185)	KOOS II (*N* = 370)	KOOS III (*N* = 303)	KOOS IV (*N* = 70)	*P*
Age	56.30 (±12.41)	58.25 (±12.44)	57.44 (±12.61)	58.11 (±14.70)	.381
Female	115 (62)	195 (53)	173 (57)	41 (59)	.191
Tumor volume	0.16 (±0.13)	0.54 (±0.34)	2.23 (±1.43)	5.45 (±2.48)	<.001*
Treatment failure rate	8 (4)	37 (10)	40 (13)	7 (10)	.017*
Mean-time-to-recurrence	5.46 (±4.14)	4.97 (±2.85)	4.52 (±2.70)	4.75 (±4.50)	.771
Paddick index	0.68 (±0.12)	0.78 (±0.12)	0.85 (±0.17)	0.85 (±0.12)	<.001*

Values are presented as the number of patients (%) unless indicated otherwise.

* Significant p-values (*P* < .05) are shown with *.

**Figure 3.**
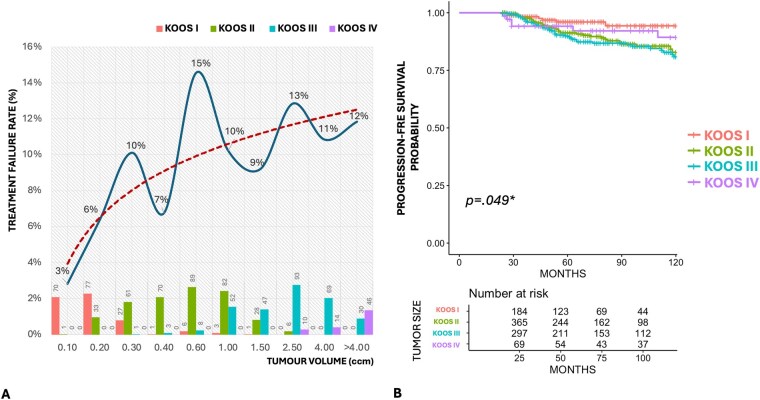
Treatment response depending on KOOS-Classification. (A) Shows the incidence of treatment failure dependent on tumor volume and the frequencies of Koos grouping dependant on the tumor volume with a logarithmic trend line (red). (B) Shows the Kaplan-Meier–analysis dependent on the Koos classification.

**Figure 4.**
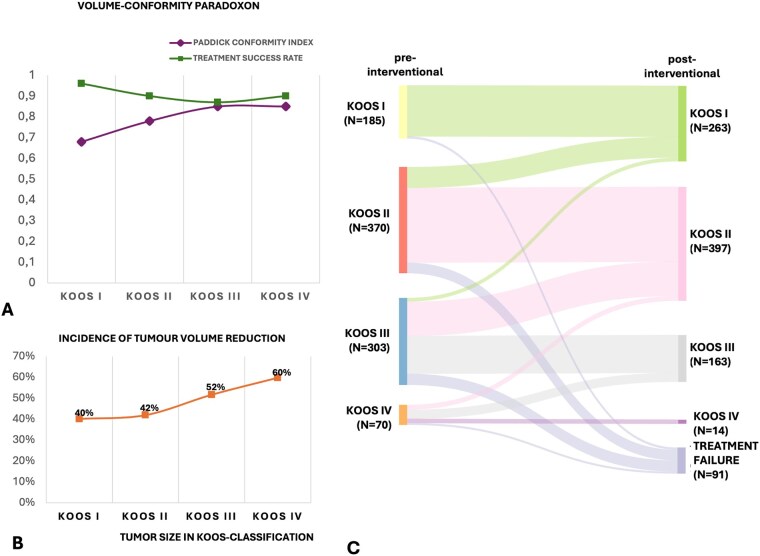
Tumor volume representation and posttreatment tumor size reduction according after treatment success or failure (in KOOS). (A) Shows the volume-conformity paradoxon. (B) Shows the rate of tumor volume reduction if treatment was successful. Treatment outcome was defined based on volumetric response (see Methods section). (C) Shows the changes in clinical size category changes in KOOS after treatment.

The treatment failure rate according to TV is shown in Figure 3A. The Kaplan-Meier–analysis on progression-­free-survival (PFS) according to KOOS classification is shown in Figure 3B. Mean time to recurrence depending on KOOS classification is shown in Table 2. The PCI was significantly higher in the treatment failure arm (*P =* .018). The behavior of PCI and treatment success rates according to tumor size in KOOS is shown in Figure 4A. Postinterventional tumor shrinkage was the highest with 60% of the initial TV in KOOS IV, 52% in KOOS III, 42% in KOOS II, and 40% in KOOS I, the proportion of tumors, which were then radiographically reclassified according to KOOS postinterventionally is shown in Figure 4B and C.

**Figure 5.**
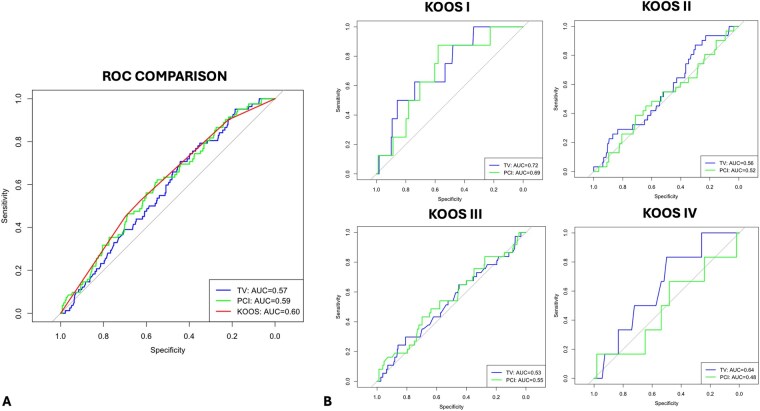
Receiver operating curves comparing model performance in predicting treatment failure. The blue curve represents the model using continuous tumor volume (TV), green represents the model using Paddick Conformity Index (PCI), and red represents the model based on KOOS classification. The *x*-axis shows the specificity and the *y*-axis the sensitivity. (A) Shows all three models in a receiver operating curve comparison. (B) Shows the predictive value of TV and PCI depending on KOOS classification with the best predictive value in KOOS I, and worse predictive value of PCI in KOOS IV tumors.

First, a model using continuous TV to predict treatment failure showed an AUC of 0.58 (95%:0.51-0.63) in the general cohort (Figure 5). The models using KOOS classification and PCI were similarly specific (KOOS: AUC of 0.60 (CI95%: 0.54-0.66), PCI: AUC of 0.59 (95%: 0.53-0.66)). The behavior of AUC of the models using TV and PCI were then compared within the KOOS classified tumors. The best predictive performance of TV (AUC of 0.72 (95%: 0.55-0.88)) and PCI (AUC of 0.69 (95%:0.53-0.86)) was present in KOOS I, but decreased in higher KOOS classes. The worst predictive performance of PCI was indeed in KOOS IV with an AUC of 0.48 (95%: 0.21-0.76).

In multivariable Cox regression, neither TV nor the PCI was independently associated with progression (HR 1.13, *P* = .20 and HR 1.50, *P* = .50, respectively). These findings indicate that PCI does not provide independent prognostic information after adjustment for relevant clinical factors (Table 3). In a separate multivariable model including KOOS classification instead of TV (to avoid collinearity), KOOS classification was also not independently associated with treatment failure (HR 1.47, 95% CI 0.70-3.08, *P* = .312). In contrast, sex remained an independent predictor of treatment failure in both models (HR 0.62, 95% CI 0.39-0.98, *P* = .043).

**Table 3.** Multivariable Cox proportional hazards model for progression-free survival

	*HR*	*95% CI*	*P*
*log_TV*	1.13	0.9; 1.35	.197
*Age*	1.00	0.98:1.02	.880
*Male sex*	0.62	0.39:0.98	.043*
*PCI*	1.50	0.46:4.91	.504

HRs and 95% CIs are shown.

*Significant p-values (*P* < .05) are shown with *.

## Discussion

The present study investigates the predictive role of pretreatment TV in determining the long-term outcomes of SRS for VS, leveraging one of the largest and most well-characterized single-center cohorts to date. The volume in ccm is the most heterogenous in groups KOOS III and IV. The rate of treatment failure was different in the different KOOS subgroups with the lowest rate in KOOS I at 4%, 10% in KOOS II, the highest in KOOS III with 13% and 10% in KOOS IV. Interestingly, the PCI was significantly higher in the treatment failure group and significantly increased with increasing tumor size indicating a high planning conformity, but with increasing tumor size, weaker predictive performance of PCI was observed.

Although TV is frequently presumed to influence treatment outcomes in VS, the evidence supporting this association has remained inconsistent.^14-23^ Prior studies have typically been constrained by heterogeneous patient cohorts, limited sample sizes, or analyses restricted to specific tumor subgroups, leading to conflicting conclusions regarding the prognostic significance of TV.^23^^,^^27^ Consequently, while the concept that larger tumors are more prone to treatment failure is often accepted, it has not yet been systematically demonstrated across the full spectrum of tumor sizes (KOOS I-IV). This article is the first to comprehensively evaluate all tumor sizes and to provide robust evidence that tumor volume is an independent determinant of treatment failure in VS. These findings clarify a long-standing uncertainty in the literature and underscore the importance of TV as a critical parameter in treatment planning and outcome assessment.

### Tumor Volume Distribution and Treatment Success/Failure

Remarkably, the age distribution was similar in all KOOS groups, with a special feature in KOOS IV: There was an accumulation of patients under the age of 30 years with KOOS IV tumors. This peak was not present in the other KOOS groups. This phenomenon of a higher incidence of younger patients with very large VS has been described in the past.^28^^,^^29^ The variability in TV across the KOOS subgroups is most pronounced in KOOS III and KOOS IV. The best PFS was observed in KOOS I tumors. The high rate of treatment success in small intracanalicular VS has also been shown in a meta-analysis carried out by Balossier et al with a mean follow-up time of 68 months in a pooled cohort of *N* = 2371 patients.^8^

An intriguing finding in the current literature is the dual observation in large VS: They are associated with both a higher risk of treatment failure and, paradoxically, a more pronounced post-treatment volume reduction when radiosurgery is successful.^14-21^ This phenomenon reflects the heterogeneity within large tumors—while some exhibit more aggressive, radioresistant behavior leading to recurrence, others are indeed responsive and capable of substantial shrinkage. This apparent contradiction reflects a statistical phenomenon rooted in subgroup variability within the large tumor cohort and may be the reason for the inflicting data presented in literature.

From a clinical perspective, our findings suggest that patients with small-volume (KOOS I-II) tumors represent optimal candidates for primary SRS, given the high tumor control rates and favorable predictive performance. In contrast, larger and more heterogenous tumors (KOOS III-IV) require more individualized treatment strategies, including intensified follow-up, careful patient counseling regarding failure risk, and consideration of multimodal approaches in selected cases.

### The Paddick Conformity Index Paradoxon

The finding that larger VSs exhibit a better PCI—a metric often used to evaluate the conformity and precision of radiosurgical dose delivery—yet show worse tumor control, highlights the complex relationship between tumor size, treatment precision, and biological behavior. A higher PCI generally reflects improved dose targeting, and is also dependent on TV.^24^ However, in the context of larger tumors, this favorable dosimetric outcome may not fully overcome the inherent challenges posed by the increased TV itself.

Wu et al examined the impact of TV and shape complexity on simulated targets and reported that the ratio of prescription isodose volume to the TV and PCI values were higher than the conformity measures for smaller and more irregular targets.^30^^,^^31^ Sümer et al confirmed this observation in a patient cohort of *N* = 234 showing that flat and nonspherical tumor shape worsened therapeutic planning conformity and showed—concordant to our study—better outcome compared to their larger, more spherical tumor counterparts with larger TV.^31^ However, their study only included a follow-up-time of 2 years. Our study stands out with a long-term follow-up of over 6 years.

The reason for the radioresistence in larger VS remains unknown, it may possibly be due to their more complex vascular architecture or greater proliferation rate, which could diminish the effectiveness of SRS.^32^ Thus, while the PCI may suggest technical efficacy in delivering radiation, it does not necessarily translate into superior biological outcomes for larger tumors.^24^^,^^30^ This underscores the need for more nuanced models that integrate volumetric, biological, and technical parameters to better predict treatment success in large VSs.

### Manner of Tumor Size Measurement

The question remains, whether KOOS is sufficient for pre-interventional measurement for VS about to be treated with SRS. The KOOS classification, originally developed to guide surgical decision-making, categorizes VSs based on their anatomical extension and brainstem compression.^10^ While it remains widely used in clinical reporting, its relevance in the context of SRS can be debated. Using KOOS in the context of pre-SRS tumor size measurement indeed is beneficial for comparability, especially when performing comparative studies or comparing treatment efficacy between surgery and SRS.^33^ Still, unlike surgical resection, SRS may be less constrained by anatomical accessibility and instead depend more on other factors, for example, tumor volume, proximity to critical structures, or radiobiological behavior. Our findings show that treatment response and failure rates generally correlate with volumetric parameters and KOOS with similar performance. However, with an overall AUC of 0.57, the predictive value of TV for treatment success or failure remains capable of improvement. Although TV showed a significant association with treatment failure, the modest predictive performance of volumetric models precludes the definition of rigid exclusion thresholds for SRS. Instead, TV should be interpreted as a continuous risk marker that supports risk stratification.

Despite its intended role as a marker of treatment precision, a higher PCI did not translate into improved tumor control in our cohort. Even after multivariable adjustment, PCI failed to demonstrate independent prognostic value, indicating that technical conformity alone is insufficient to overcome the biological and volumetric challenges of larger VSs.

Interestingly, among those tumors that responded successfully to SRS, the magnitude of long-term postinterventional volume reduction was the largest in larger tumors, suggesting that even high-volume lesions can achieve regression when appropriately selected for radiosurgical treatment. The continuous nature of TV could potentially allow for identifying threshold values beyond which the likelihood of treatment failure significantly increases. Establishing such volumetric cutoffs could enable clinicians to better distinguish between low-risk and high-risk patients and to tailor treatment intensity or follow-up protocols accordingly.

### Other Prognostic Parameters and Outlook

The rate of treatment failure was significantly higher in females compared to males, which represents a recently found particular difference in treatment response based on biological sex, confirmed also by this current patient cohort.^3^^,^^5^^,^^34^ Predicting which tumors are likely to respond favorably to SRS remains a critical unmet need. Incorporating additional pretreatment variables—such as growth kinetics, enhancement patterns on MRI, or radiomic features—may improve predictive accuracy for treatment outcomes. Future studies should aim to integrate volumetric data with biological and imaging biomarkers to develop validated predictive models that guide personalized treatment planning and optimize long-term tumor control. Future studies should further include longitudinal volumetric response patterns to evaluate the impact of pretreatment TV on SRS treatment response. While prolonged pseudoprogression remains actively debated and is not yet reflected in clinical guidelines, careful validation is warranted, as the long-term outcomes of patients with continued tumor growth without formal treatment failure remain incompletely understood in these studies.^34^^,^^35^

### Limitations and Strength of This Study

This study highlights the importance of excluding short-term follow-up cases, particularly to avoid misclassifying pseudoprogression as treatment failure. Our strict inclusion of patients with a minimum of 2 years of radiographic follow-up enhances the reliability of our results and may better distinguish true progression from transient, benign post-radiosurgical changes. On the other hand, excluding patients with less than 2 years of follow-up to account for pseudoprogression may introduce survivorship bias and lead to an underestimation of early true progression. Because follow-up duration varied substantially, the presented volumetric and Koos class transitions represent time-dependent observations rather than uniform longitudinal trajectories, which limits comparability between individuals. While the data were derived from a prospectively maintained registry, some relevant data were also collected retrospectively. Due to the length of study interval and also patients treated in the early 2000s, the slice thickness and MRI quality may have varied in the interval of almost 20 years—in which the study cohort was treated. Interrater agreement between the two neurosurgeons was not formally assessed, which represents a limitation of this study. However, the large sample size and long-term follow-up strengthen the external validity and clinical relevance of our findings.

## Conclusion

This study demonstrates a clear correlation between initial tumor size and treatment outcomes in patients with VS undergoing SRS. The rate of treatment failure increases with TV and size, underscoring the impact of tumor size on treatment failure. Our finding of the Paddick Paradox underscore the complexity of treating large VS with SRS. While dosimetric parameters such as the PCI are important for planning, they may not fully capture the biological behavior of larger tumors. In small tumors, their predictive performance was rather good, but decreased in larger KOOS size classes. This highlights the need for a multifaceted approach that considers both dosimetric and biological factors when predicting treatment outcomes for individualized treatment strategies.

## Data Availability

All data and materials are available and can be provided upon reasonable request.
